# Genome-wide identification and expression analysis of *NPR1-*like genes in pearl millet under diverse biotic and abiotic stresses and phytohormone treatments

**DOI:** 10.1080/15592324.2025.2552895

**Published:** 2025-09-07

**Authors:** Jagatjeet Nayak, Chanwala Jeky, Baisista Saha, Nrisingha Dey, Soumya Ranjan Mahapatra, Namrata Misra, Mrunmay Kumar Giri

**Affiliations:** a School of Biotechnology, Kalinga Institute of Industrial Technology (KIIT) Deemed to be University, Bhubaneswar, India; b Plant Biotechnology, Institute of Life Sciences, Bhubaneswar, India; c Plant Biotechnology, Ramakrishna Mission Vivekananda Educational and Research Institute, Kolkata, India

**Keywords:** Nonexpressor of pathogenesis-related 1, systemic acquired resistance, downy-mildew, salicylic acid, methyl jasmonate, biotic, abiotic, pearl millet

## Abstract

Nonexpressor of pathogenesis-related genes 1 (NPR1) is a master regulator of salicylic acid (SA)- facilitated plant hormone signaling and plays a crucial role in plant defense through the activation of systemic acquired resistance (SAR). Although *NPR1-*like genes are associated with stress responses in a variety of plant species, no thorough genome-wide investigation of these genes has been undertaken in pearl millet (*Pennisetum glaucum*). This study discovered seven *PgNPR1*-like genes on four pearl millet chromosomes (Chr1, Chr2, Chr4, and Chr6), which exhibit close affinity to NPRs from other plants and have common gene structures, conserved motifs, and domains. The promoter regions of *PgNPR1-*like genes have numerous cis-acting elements connected with biotic and abiotic stresses, natural plant growth, and development. The qPCR results showed that *PgNPR1*-like genes were differentially expressed in distinct tissues, developmental stages, and under various biotic and abiotic stresses. Some putative *NPR1-like* genes, such as *Pgl_GLEAN_10029279*, *Pgl_GLEAN_10004488*, *Pgl_GLEAN_10004489*, and *Pgl_GLEAN_10015079*, showed considerable expression in response to abiotic stimuli such as heat, drought, and salinity. The *PgNPR1-*like gene *Pgl_GLEAN_10029279* was observed to be differently expressed upon treatment of hormones such as SA and MeJA. *Pgl_GLEAN_10029279* was also significantly expressed after *Magnaporthe grisea* infection, which causes blast in pearl millet. *In silico* expression study of the *PgNPR1-*like genes after *Sclerospora graminicola* infection, causing downy-mildew disease, revealed that *Pgl_GLEAN_10029279* and *Pgl_GLEAN_10004489* were significantly upregulated. In addition, the docking results also showed that Pgl_GLEAN_10029279 and Pgl_GLEAN_10007810 out of all seven PgNPRs have strong interactions with the ligand SA, which proves their potential involvement in SA signaling and hence plant defense. These results offer a firm framework for comprehending the roles and development of *PgNPR1-*like genes in pearl millet.

## Introduction

Pearl millet (*Pennisetum glaucum*) is a drought-tolerant crop that thrives in arid climates and drylands. It can withstand severe abiotic environments such as high soil pH, dryness, and extreme temperatures.^1^ Pearl millet offers a higher nutritional value, comprising vital micronutrients such as iron and zinc. Pearl millet has a good protein content of up to 19%, high fiber, low starch, and other minerals.​​​​​​^2^^,^^3^​​​​​​ Despite having all these benefits, pearl millet is subjected to numerous biotic and abiotic challenges. Biotic stresses encompass bacterial pathogens, viral diseases, fungal infections, and infestations by a diverse range of insects. Whereas plants also confront abiotic challenges such as salt and heat, which impact their growth, development, and food yields, yet they have adaptable systems. The cultivation of robust crops is critical in the face of soil salinity and global warming. In the face of climate change, pearl millet has great promise for sustainable agriculture and food security because of its nutritional richness and climate resiliency.

Plants have developed an extremely complex and potent innate immune system to fight against many pathogens, such as bacteria, fungi, viruses, and oomycetes.^4-6^ The initial line of defense against pathogen threats is known as pathogen-associated molecular pattern (PAMP)- triggered immunity (PTI), and it starts on the plant cell surface.^7^^,^^8^ PTI responses are triggered by pattern-recognition receptors (PRRs) in the plasma membrane, which identify PAMPs such as lipopolysaccharides (LPS), bacterial flagellin (flg22), and fungal chitin.^9-11^ On the other hand, many microbial pathogens release effectors during infection, which might cause effector-triggered susceptibility (ETS) and decrease PTI.^12-14^ Plants have evolved a second layer of locally induced resistance called effector-triggered immunity (ETI) along their evolutionary history.^15^^,^^16^ By identifying these effectors in particular to the adversary, plant intracellular sensors encoded by resistance (R) genes start ETI responses. R gene- mediated defenses, sometimes referred to as the hypersensitive response (HR), offer robust resistance and successfully impede pathogen development by inducing programmed cell death (PCD). Salicylic acid (SA), a plant defense hormone, is produced and accumulates as a result of this local immune response in both infected and uninfected tissues. This leads to the deployment of systemic acquired resistance (SAR) after HR throughout the plant. Broad-spectrum, persistent systemic resistance to secondary infections is provided by SAR, which is defined by the expression of many antimicrobial pathogenesis-related (PR) genes in all plant tissues.^17-19^*Arabidopsis* mutants that are defective in establishing an SA-mediated defensive response led to the discovery of NPR1, a master regulator of the SAR pathway.^20^

Studies have reported that NPR1 positively regulates the SAR-mediated defense response in plants. The transcription coactivator NPR1 functions as an SA receptor.^21^^,^^22^ The BTB/POZ domain (Broad Complex, Tramtrack, and Bric a Brac/ Pox virus and Zinc finger) and ankyrin repeat domain (ANK) are two protein‒protein interaction domains that are encoded by the *NPR1* gene.^23^^,^^24^ The ankyrin repeat domain links to the TGA motif present in the bZIP transcription factor, while the BTB/POZ domain promotes the dimerization of NPR1.^23^^,^^25^ In addition to these domains, it has a nuclear localization region and a transcriptional activation motif in the C-terminal.^26^^,^^27^ SA levels in the plant are low before pathogen invasion, and NPR1 localizes as a dormant oligomer in the cytoplasm via disulfide bonds. Upon pathogen attack, the plant mounts a defense response characterized by an increase in endogenous SA levels. This elevation in SA triggers a redox signaling cascade within the cytoplasm. Subsequently, NPR1 undergoes a conformational change, transitioning into an activated monomeric state and translocating from the cytoplasm to the nucleus through the nuclear pore. As a transcriptional coactivator, NPR1 interacts specifically with TGA TFs, exerting regulatory control over the expression of downstream target genes.^28^ Then, *pathogenesis-related* (*PR*) gene expression increases, inducing SAR.^29^^,^^30^ Although NPR1 is well known for its role under biotic stress conditions in plants specifically to pathogens, NPR1 is also effective on various abiotic stresses.^31^^,^^32^ These affect plant immunity mainly through reactive oxygen species (ROS) production and SA signaling.^33^

 In numerous plants, including tomatoes, avocados, strawberries, and bananas, *NPR1-*like genes are crucial for disease resilience, tissue growth, and organ development.^34-37^ There is a lack of research investigating the roles and characteristics of *NPR1-*like genes in pearl millet, which could provide valuable insights. The *NPR1-*like gene family members in the pearl millet genome were screened and analyzed for the first time in this research. Furthermore, the investigation included the examination of the phylogenetic analysis, gene structure, conserved motif pattern, cis-regulatory elements, chromosomal distribution, and tissue-specific expression profiles of *NPR1-*like genes in *P. glaucum*. We also showcased the expression of *PgNPR1*-like genes in response to SA, MeJA treatment, and infection with *Sclerospora graminicola* and *Magnaporthe grisea*. In particular, *Pgl_GLEAN_10029279* was observed to have significant expression upon treatment with SA, MeJA, and after infection with both pathogens. Expression profiling of the *PgNPR1*-like genes under abiotic stress, such as heat, salinity, and drought, was also performed. The findings presented here establish a potential framework for future functional investigations of *PgNPR1-*like genes along with their genetic improvements in pearl millet.

## Materials and methods

### Exploration of NPR1 proteins in *P. glaucum* through *in silico* analysis

By utilizing reference protein sequences containing the *NPR1-*like gene domain from wheat, sorghum, rice, barley, and Arabidopsis, a search was conducted to identify PgNPR proteins in *P. glaucum*. Pearl millet proteome sequences were obtained from the pearl millet genome database, and 252 reference sequences were obtained from Phytozome v12.1.6, Plant Genomics Resource.^38^ The Arabidopsis Information Resource (TAIR) (https://www.arabidopsis.org/).^39^ and Oryzabase: an integrated biological and genome information database for rice.^40^ The alignment of the reference sequences was performed using Clustal Omega, and the consensus of the aligned sequences was used to generate the HMM profile.^41^ The HMM profile for the aligned reference sequences was created using the HMMER tool v3.2 with default parameters, considering the degree of conservation among the sequences.^42^ A search using HMMER was carried out against the proteome database using the generated NPR1 profile to find possible homologous NPR1 sequences in *P. glaucum*. Furthermore, the presence of the NPR1 domain in the identified potential NPR1 homologs was validated by performing an HMMER scan, SMART, and NCBI-CD search using the *PFAM* domain (PF12313.7).

### Phylogenetic tree creation and analysis of sequences

The NPR1 sequences of pearl millet, rice, Arabidopsis, wheat, barley, and sorghum were aligned with default parameters using MUSCLE.^43^ It was then imported into MEGA v7.0 software.^44^ To construct the phylogenetic tree, the maximum likelihood method was used with the bootstrap test method for 1000 replications. Substitution model: Jones–Taylor–Thornton (JTT) model; rates among sites: gamma distributed (G) and gaps/missing data treatment: partial deletion.^45^ The physiochemical characteristics of each PgNPR1-like protein sequence, such as amino acid composition, isoelectric point, instability index, molecular weight, hydropathicity, aliphatic index, and subcellular localization, were analyzed using the ProtParam program available on the ExPASy server.^46^

### Chromosome mapping and gene structure analysis

The chromosomal coordinates with starting and end points were loaded into MapInspect v1.0 software (http://mapinspect.software informer.com/) in ascending order to visualize the chromosomal location of each *NPR1*-like gene. The physical map of the *NPR1*-like genes was generated using their chromosomal positions on all seven chromosomes. From the pearl millet genome library (http://cegsb.icrisat.org/ipmgsc/), the coding sequences of the identified *NPR1-*like genes were obtained, and using the accessible genomic coordinates, the genomic sequences of each *NPR1-*like gene were extracted.^47^ By utilizing the coding and genomic sequences in the GSDS web server v2.0, the intronic and exonic locations of each *NPR1-*like gene in *P. glaucum* were determined.^48^ MEME software v5.1.0 was used to search for conserved motifs in these *NPR1*-like genes.^49^

### Identification of cis-regulatory elements

The Plant CARE database was used to investigate the presence of cis-regulatory elements in the 1.5kb upstream promoter region of pearl millet's *NPR1-*like genes.

### Structural prediction and molecular docking

The Robetta server has been used to generate 3D models for two protein sequences of Pearl Millet sequence, viz., Pgl_GLEAN_10007810. Furthermore, the PROCHECK, VERIFY 3D, and ERRAT servers (https://saves.mbi.ucla.edu/) validated each prediction model. The 3D structure of the ligand salicylic acid (PubChem CID:338) in SDF format was retrieved from PubChem (https://pubchem.ncbi.nlm.nih.gov/) and converted into 3D structures in PDB format through the PyMOL tool. The AutoDock Vina was used with its default parameters for the docking environment. The PyMOL visualization tool analyzes the optimum docked conformation with the maximum binding energy.

### Plant materials and treatments

Pearl millet cultivar (7042S and IP3757) seeds were obtained from the International Crops Research Institute for the Semi-Arid Tropics (ICRISAT), Patancheru, Hyderabad, Andhra Pradesh, India. The seeds were sown in the soil mixture containing pots, where the soil and vermiculite were mixed at a volume ratio of 4:1.^50^ Then the plants were allowed to grow under long-day conditions of 16/8-h light and dark periods at a temperature of 25°C (±2). Pearl millet plants from different time points and developmental stages were harvested from different tissues, such as flag leaves, roots, stems, inflorescences, seeds, and mature leaves, under usual growth conditions. For the salinity stress experiment, 4-week-old plants were rinsed, immersed in 250mM NaCl solution and kept in distilled water for the control treatment. Samples were taken after 12 h of treatment. After the fourth week of pearl millet seed sowing, irrigation was stopped for 6 d, resulting in a drought state. In the meantime, the control plants continued to receive regular watering on alternate days. Irrigation was applied to the treated and control plants on the seventh day to aid in their recovery. At days 1, 5, 7, and 11, the leaves of the treated and control plants were collected accordingly. Heat stress condition was created by giving heat treatment at 42°C. Leaf samples were collected after 3 and 12 h of treatment. After foliar spray of 0.1 mM of SA and MeJA along with 0.1% Tween 20, leaf samples were collected at 0, 2, 12, 24, and 48 h. 3- and 5-d post-SA and MeJA treatment samples were also taken. For the fungal spore treatment, the pearl millet plants were sprayed with spore suspension (adjusted to 12 × 10^4^ spores/ml) with 0.1% Tween 20, and the control plants were treated with only 0.1% Tween 20.^51^ Both the infected and control plants were maintained under 100% relative humidity. The leaf samples were taken at 0, 3, and 5 d after infection. To guarantee variety and representativeness, samples were obtained from several leaves of distinct plants for every biological triplicate. In particular, leaves from several plants in the experimental group were randomly chosen at similar developmental stages.

### Relative quantification of *PgNPR1-*like gene expression

To choose potential *NPR1-*like genes from pearl millet, the phylogenetic, sequence identity, homologous pairing, and alignment results of all the *NPR1-*like genes were compared with those of the well-characterized *NPR1-*like genes of Arabidopsis, wheat, rice, barley, and sorghum. To determine how these chosen *NPR1-*like genes vary in their expression patterns, the transcript level of these genes was examined. The Primer3 tool (http://primer3.ut.ee/) was used to design primers.^52^ In the optimization process, the genomic DNA of pearl millet was employed as a template for PCR.

Total RNA was isolated manually with a standardized laboratory protocol using TRIzol from frozen plant tissues and further analyzed using a Nanodrop 2000 spectrophotometer (Eppendorf AG-22331) for concentration and purity. DNase I (Thermo Scientific™-EN0525) digestion was performed on 100 ng of RNA samples, and the cDNA synthesis kit (Verso cDNA Synthesis Kit-Thermo Scientific™-AB1453A) was used to convert the RNA to cDNA. Initially, semiquantitative RT‒PCR was performed for 35 cycles to check the integrity of the prepared cDNA. The quantitative PCR was done using the ABI StepOne Real-Time PCR System. A total of 20.0  µl of the reaction mixture was prepared containing 10.0 µl of PowerUp™ SYBR™ Green Master Mix (Applied Biosystems™-A25742), 0.4 μl of each forward and reverse primer (10 μM), 4.0 μl (10 ng) of cDNA, and the volume was adjusted with nuclease-free water. NTC (nontemplate control) mixtures were also taken, having all the elements except the cDNA template. *GAPDH* was taken as an endogenous control for all the experimental sets. To achieve an average number with the appropriate standard deviation, triplicate biological samples for each reaction were run. The primer sequences and the PCR conditions are given in the supplementary files (Supplementary Table S3).

### *In silico* prediction of *PgNPR1* expression upon downy-mildew infection

Initial information was collected from the supplementary data provided in a previous transcriptomic study of downy-mildew infection in Pearl millet resistant and susceptible varieties.^53^ We downloaded the sequences of reported Sequence Read Archive (SRA) files, viz. SRX885597, SRX1885493, SRX1885494, and SRX1885495 from the SRA database of the NCBI (www.ncbi.com). Using BioEdit 7.2, all the PgNPR1-like sequences were blasted with the SRA sequences from the database as mentioned above.^54^ Then the sequence similarities were observed using those BLAST results containing SRA hits.

## Results

### Identification of *NPR1-like* genes in pearl millet

The pearl millet database (http://cegsb.icrisat.org/ipmgsc/), containing 38,579 proteome sequences, was used to screen for orthologs of wheat, sorghum, rice, barley, and Arabidopsis *NPR1-*like gene family proteins in pearl millet. Hidden Markov model (HMM) profiling was used to look for *NPR1-*like genes. The HMMER search revealed a total of 252 comparable sequences. The existence of typical ankyrin repeats, as well as BTB and NPR1-like C domains, was manually searched in these identified PgNPR1-like sequences. It was identified that *P. glaucum* contains a total of seven PgNPR1-like sequences based on the presence of a maximum number of domains (Supplementary Table S1).^51^ The conserved amino acid sequences were visualized using a color scale ranging from blue to red, where blue represents regions with the lowest conservation and red represents highly conserved sequences (Supplementary Figure S1). From this conservation, it was found that all seven identified putative NPR1-like proteins contained an *N*-terminal BTB/POZ domain and ankyrin repeats (Ank) in the central position. In the C-terminal region, two regions were found, named NIMIN 1/2 binding region and nuclear localization signal (NLS). The *in silico* features, such as amino acid length, which ranged from 362 amino acids (Pgl_GLEAN_10015079) to 662 amino acids (Pgl_GLEAN_10004488), isoelectric point (pI) ranged from 5.16 (Pgl_GLEAN_10004488) to 6.53 (Pgl_GLEAN_10015079), and protein molecular weight (MW) ranged from 39.108 kDa (Pgl_GLEAN_10015079) to 73.264 kDa (Pgl_GLEAN_10004488), were enumerated. Furthermore, *in silico* prediction for subcellular localization revealed that all the PgNPR1-like proteins are localized in the cytoplasm and nucleus, and some of them, i.e., Pgl_GLEAN_10004488, Pgl_GLEAN_10004489, Pgl_GLEAN_10007810, Pgl_GLEAN_10029279, and Pgl_GLEAN_10033256, in chloroplast (Supplementary Table S2).

### Phylogenetic analysis

**Figure 1.**
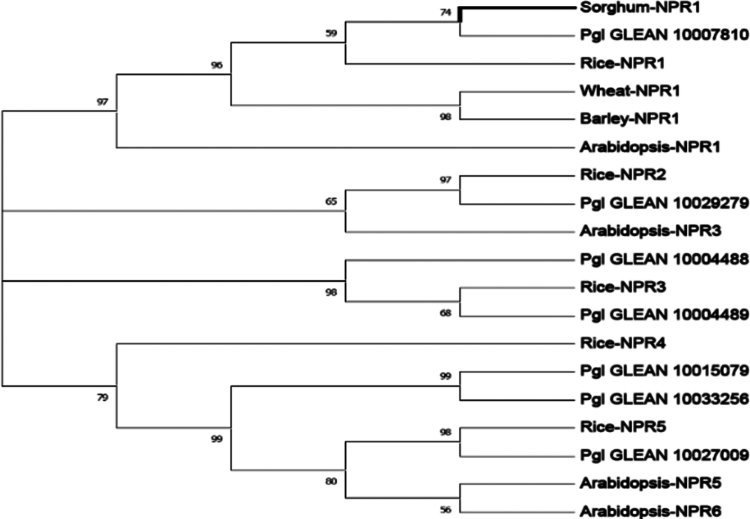
The phylogenetic representation of PgNPR1-like sequences with Arabidopsis, rice, sorghum, wheat, and barley. All sequences were aligned by MUSCLE, and a phylogenetic tree was constructed by MEGA v7.0 using the maximum likelihood method with 1000 bootstrap replications.

A phylogenetic analysis was conducted to examine the evolutionary relationship of different NPR1-like members of pearl millet with Arabidopsis and monocot plants, such as rice, wheat, barley, and sorghum. The phylogenetic tree revealed that all the putative NPR1-like sequences of pearl millet share common ancestors with the other NPR sequences, including NPR1, NPR2, NPR3, and NPR5 of other plants. It also revealed that some of the putative PgNPR1 sequences showed close homology with other reported NPRs taken as reference sequences. We found that two of the PgNPR1-like sequences, viz. Pgl_GLEAN_10007810 and Pgl_GLEAN_10029279 have the closest homology with sorghum-NPR1 and rice-NPR2, according to their shared clade in the phylogenetic tree (Figure 1).

### Chromosomal distribution and structure analysis of *PgNPR1-*like genes

**Figure 2.**
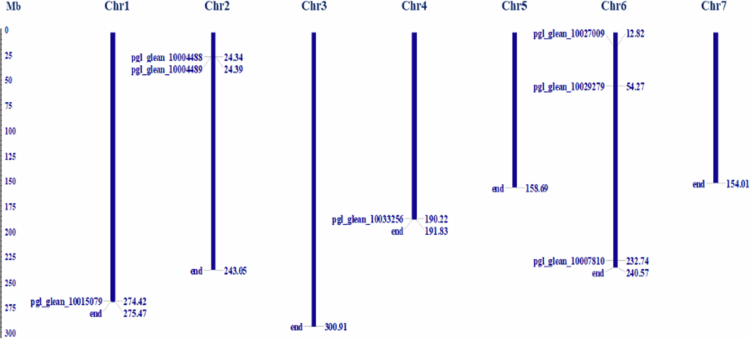
The chromosomal distribution and positioning of PgNPRs across all seven chromosomes of pearl millet. Seven chromosomes with varying lengths are shown on the Mb (million base pair) scale on the left, where individual chromosomes (bars) are labeled with their respective *PgNPR1*-like genes.

Seven putative *PgNPR1-*like genes were randomly distributed across four of the seven chromosomes in the *P. glaucum* genome (Figure 2). The majority of *PgNPR1-*like genes were identified on the 2nd (two genes) and 6th (three genes) chromosomes, with the least on the 1st and 4th (one gene each). However, chromosomes 3, 5, and 7 do not have *NPR1-*like genes. Three *PgNPR1-*like genes were located at chromosomes 1, 4, and 6, limited to the telomere regions.

**Figure 3.**
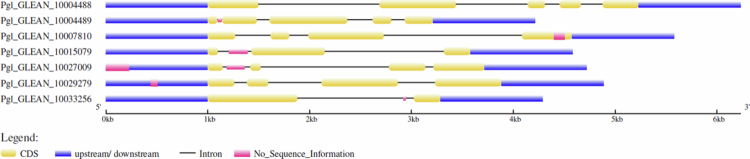
The structural analysis of identified *PgNPR1-*like genes using the GSDS web server v2.0. The structural features of the genes are represented in different colors as described in the figure.

 Prediction of intronic/exonic locations and 5′/3′ untranslated regions (UTR) of each *PgNPR1-*like gene was performed by leveraging the coding and genomic sequences through the GSDS web server v2.0. Intronic sequences were observed in all of the putative genes. Each of the three genes has a maximum of five exons (Figure 3).

**Figure 4.**
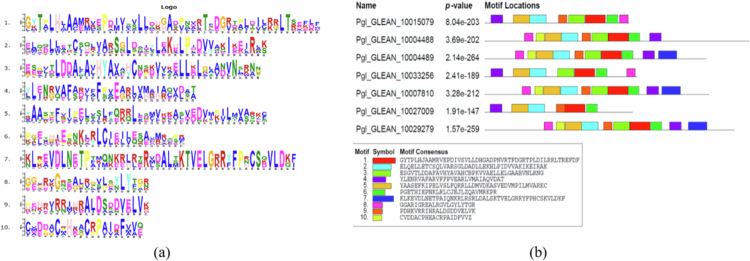
(a) Conserved amino acid residues are present in the identified motifs. (b) Representation of the conserved motif location in all the putative *PgNPR1-*like genes.

The MEME suite was used to search for conserved motifs in potential NPR1-like proteins (Figure 4a). In PgNPR1-like proteins, we have identified the existence of ten conserved motifs. Three out of the seven NPR1-like proteins include all ten conserved motifs. Motif numbers 1, 2, 4, 5, and 6 are found in all seven of the putative PgNPR1 proteins; however, motif 7 is found in only three of them (Figure 4b).

### Cis-regulatory elements analysis

**Figure 5.**
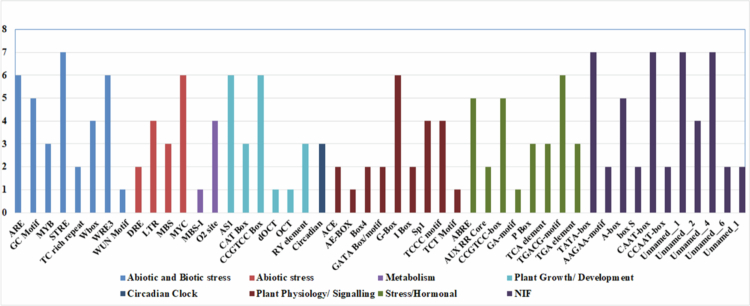
*In silico* analysis of cis-regulatory elements: the frequency of identified putative cis-acting elements in the 1.5 kb upstream region of *PgNPR1-*like genes. The color on the bars indicates the class (shown in the legend) to which it belongs. The Y-axis represents the number of *PgNPR1-*like genes (out of seven) that have the specific type of cis-regulatory elements mentioned on the X-axis.

The PlantCARE database was used for the promoter analysis of *PgNPR1-*like genes by taking a 1.5 kb upstream region. Among all the putative *PgNPR1-*like genes, a collective of 61 cis-regulating elements (CREs) were observed. Some of them were found to be specifically involved in abiotic and biotic stress, such as ARE, MYB, STRE, and WRE3. Interestingly, cis-elements related to SA responsiveness (TCA element), MeJA responsiveness (TGACG motif), and biotic stress-related (W-box, TC-rich repeats, and TCA element) were present in the identified PgNPR members, which indicates their involvement in the biotic stress responses of pearl millet. Other cis-regulating elements were also present, having specific roles in processes, such as plant metabolism, growth and development, physiology, signaling, circadian clock, and NIF (nitrogen fixation) (Figure 5).

### Structural prediction and molecular docking

**Figure 6.**
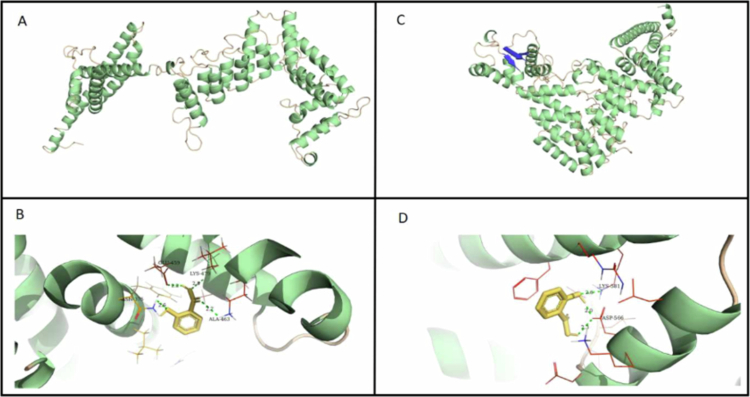
Homology modeling and molecular docking of PgNPRs. (A) The 3D model for Pgl_GLEAN_10007810 and (C) the 3D model for Pgl_GLEAN_10029279. The molecular docking with salicylic acid is shown in (B) and (D) for Pgl_GLEAN_10007810 and Pgl_GLEAN_10029279, respectively.

The three-dimensional (3D) protein structure provides vital information regarding protein function, enabling successful experimentation to be developed. The Robetta server has been used to generate 3D models for two protein sequences of pearl millet sequences (Figure 6A,C) viz., Pgl_GLEAN_10007810, showing close homology with NPR1 of rice, wheat, sorghum, etc., and Pgl_GLEAN_10029279, which is closest to rice NPR2 (Figure 1). Furthermore, the PROCHECK, VERIFY 3D, and ERRAT servers (https://saves.mbi.ucla.edu/) validated each prediction model. The investigation shows that 90.2% and 88.7% of the residue was found in the favored regions, corresponding to Ramachandran's plots of Pgl_GLEAN_10007810 and Pgl_GLEAN_10029279. In addition, two such models were reliable, as they had little and no residue in the disallowed region. The overall quality factor in the predicted 3D organism structures was >95, as shown by the ERRAT server results. VERIFY3D (data not shown here) shows the total precision of the model structures.

Molecular docking is a method used to predict the structure and spatial orientation of interacting proteins/ligands and the underpinning binding residues. Given that *AtNPR1* is supposed to have a binding affinity with SA, we attempted to dock SA with Pgl_GLEAN_10007810 and Pgl_GLEAN_10029279. The 3D structure of the ligand salicylic acid (PubChem CID:338) in SDF format was retrieved from PubChem (https://pubchem.ncbi.nlm.nih.gov/) and converted into 3D structures in PDB format using the PyMOL tool. AutoDock Vina was used with its default parameters for the docking environment. The PyMOL visualization tool analyzes the optimum docked conformation with the maximum binding energy. The residues involved in the interaction of the ligand salicylic acid with the Pgl_GLEAN_10007810 protein were found to be ASN 375, GLU 459, LYS 479, and ALA 463 with distances of 2.6, 2.2, 2.8, and 2.7 Å, respectively (Figure 6B). Similarly, LYS 581 and ASP 566 were observed to play a vital role in the interaction of SA with Pgl_GLEAN_10029279 (Figure 6D).

### Relative expression analysis of *PgNPR1*-like genes

**Figure 7.**
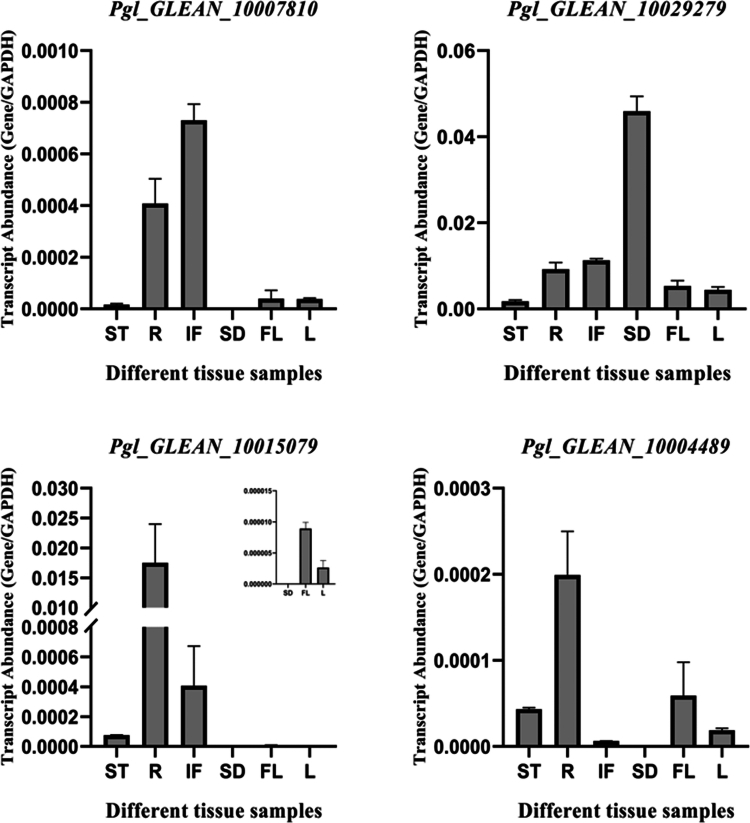
The transcriptional expression pattern of *NPR1*-like genes in *P. glaucum* across various tissues (ST: stem, R: root, IF: inflorescence, SD: seed, FL: flag leaf, L: mature leaves). The transcriptional expression patterns of *PgNPR1*-like genes was analyzed by real-time quantitative PCR. *GAPDH* was used as the internal reference gene. The data shown are representative of three independent biological replicates, and all data points indicate the mean ± standard error (SE) of the three biological repeats.

*PgNPR1*-like genes were found to be expressed in various tissues, such as stems, roots, inflorescences, seeds, flag leaves, and mature leaves; however, their levels varied considerably (Figure 7). Among all*, Pgl_GLEAN_10029279* had relatively stable expression levels detected in all the tissues and the highest in the seed samples. *Pgl_GLEAN_10015079*, *Pgl_GLEAN_10004489*, and *Pgl_GLEAN_10007810* were observed to be strongly expressed in the root samples but weakly expressed in the seed samples. The expressions of *Pgl_GLEAN_10004489* and *Pgl_GLEAN_10015079* were comparatively higher in the stem tissues. *Pgl_GLEAN_10004489* had the highest expression in flag leaf tissues, among other genes. In the inflorescence tissues, considerable expression was observed for *Pgl_GLEAN_10007810* and *Pgl_GLEAN_10015079*.

**Figure 8.**
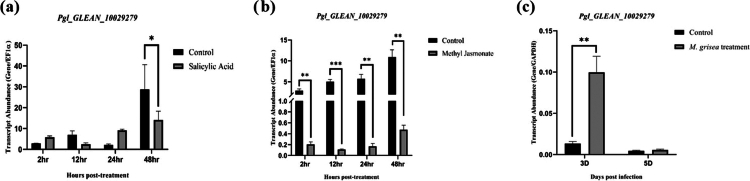
The transcriptional expression patterns of *Pgl_GLEAN_10029279* under (a) salicylic acid treatment, (b) methyl jasmonate treatment, and (c) *M. grisea* infection. The transcriptional expression pattern was analyzed by RT–qPCR. *EF1α* and *GAPDH* were used as the internal reference genes. The data shown are representative of three independent biological replicates, and all data points indicate the mean ± standard error (SE) of three biological repeats. A significant difference in mean between the samples for a given set is indicated by *P < 0.05, **P < 0.01, and ***P < 0.001 as obtained by *t*-test.

 The selected *PgNPR1-like* genes were analyzed using qRT–PCR to determine their expression pattern under foliar application of phytohormones such as SA and MeJA and after infection with the pearl millet blast causing pathogen *M. grisea*. As shown in the figure, *Pgl_GLEAN_10029279* had varying expression levels at various time periods. Specifically, under SA treatment, we observed varied transcript accumulation of *Pgl_GLEAN_10029279* (Figure 8a). Whereas, significant downregulation was observed compared to control samples when plants were treated with MeJA (Figure 8b). After infection with *M. grisea*, only *Pgl_GLEAN_10029279* had expression among all the other seven *PgNPR1-*like genes. After 3 d and 5 d, post-treatment with SA and MeJA, there was no significant expression compared to the control plants (Supplementary Figure S3). *Pgl_GLEAN_10029279* showed significant upregulation after 3 d of treatment with *M. grisea* infection (Figure 8c). The symptom of *M. grisea* infection was observed on day 5. Second, we detected transcripts of *Pgl_GLEAN_10007810* only in the semiquantitative analysis under SA and MeJA treatments (Supplementary Figure S2). In addition, no transcripts were detected for *Pgl_GLEAN_10015079, Pgl_GLEAN_10004489, Pgl_GLEAN_10004488, Pgl_GLEAN_10033256, and Pgl_GLEAN_10027009* in semi-quantitative as well as quantitative RT‒PCR analysis upon both SA and MeJA applications and *M. grisea* infection.

### *In silico* prediction of *PgNPR1* expression upon downy-mildew infection

**Figure 9.**
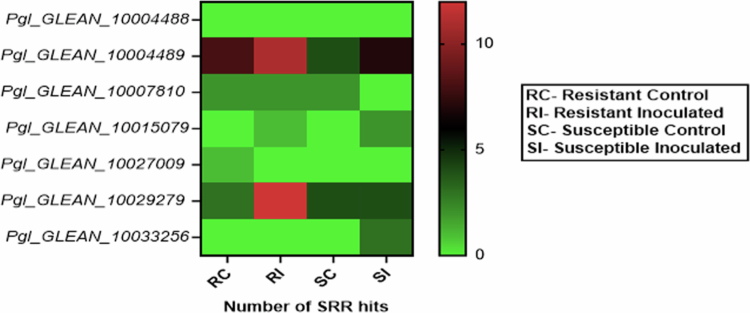
The Heatmap of transcriptomic expression profile of the *PgNPR1-like* genes upon downy-mildew infection.

To check for the potential involvement of the putative *PgNPR1-like* sequences in response to pathogen infection, we considered a previous study by Kulkarni et al.^53^ in which transcriptome sequencing of the pearl millet varieties was performed. SRR files (SRX885597, RX1885493, SRX1885494, and SRX1885495) of control and treated samples were used to identify differentially expressed *PgNPR1-like* genes upon downy-mildew infection. In RNA-Seq data analysis, specifically, we found substantial upregulation of Pgl_GLEAN_10029279 in the resistant varieties, whereas Pgl_GLEAN_10004489 showed significant upregulation in both the resistant and susceptible varieties. In addition, the differential expression pattern of other PgNPRs showed their probable involvement in the stress response to downy-mildew infection (Figure 9).

**Figure 10.**
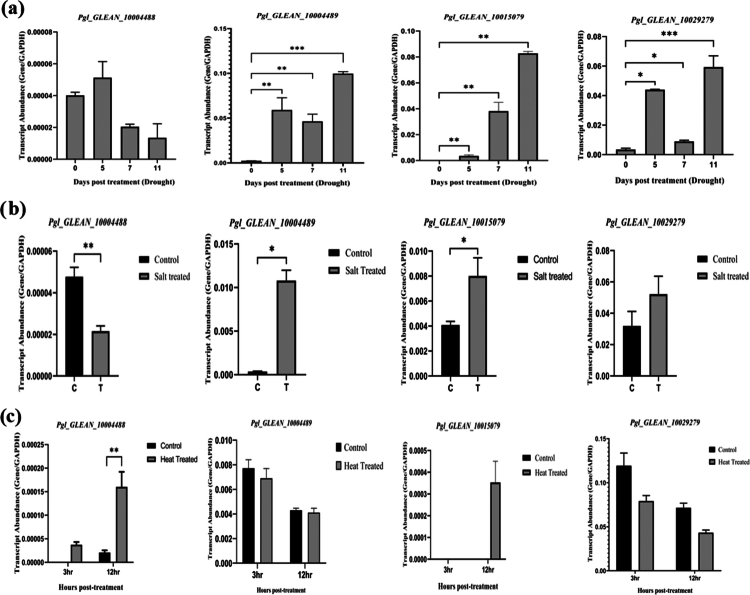
RT‒qPCR-based differential expression analysis of *PgNPR1*-like genes under various abiotic stresses, i.e., (a) drought stress (0, 5, 7, and 11 d), (b) salinity stress (12h), and (c) heat stress (3 h and 12 h). The transcriptional expression pattern was analyzed by real-time quantitative PCR. GAPDH was used as the internal reference gene. The data shown are representative of three independent biological replicates, and all the data points represent the mean ± standard error (SE) of three biological repeats. A significant difference in the means between the samples for a given set is indicated by *P < 0.05, **P < 0.01, and ***P < 0.001 as obtained by *t*-test.

The expression of *PgNPR1-*like genes under various abiotic stress conditions, such as heat, drought, and salinity, has been reported. Under heat treatment, *Pgl_GLEAN_10029279* and *Pgl_GLEAN_10004489* showed mere downregulation but *Pgl_GLEAN_10004488* showed significant upregulation (Figure 10a). A few of the potential genes showed significant expression pattern among all the seven *PgNPR1-like* genes. *Pgl_GLEAN_10029279*, *Pgl_GLEAN_10004488*, *Pgl_GLEAN_10004489*, and *Pgl_GLEAN_10015079* were observed to have differential expression after drought and salinity stress conditions. *Pgl_GLEAN_10029279*, *Pgl_GLEAN_10004489*, and *Pgl_GLEAN_10015079* showed significant upregulation to drought and salt stress, while, in contrast, *Pgl_GLEAN_10004488* showed downregulation (Figure 10b,c).

## Discussion

In this study, we have identified seven NPR1-like proteins in *P. glaucum* genome. Numerous NPR1-like proteins have already been identified and reported for various plant species, such as 9 in Chinese pear, 19 in oilseed rape, 17 in Bread wheat, 6 in wild strawberry, 5 in rice, and 6 in Arabidopsis.^55-60^ This study assessed the *NPR* family genes in pearl millet for the first time, laying the groundwork for future research on the function of PgNPR1-like proteins.

A comparison study with other reported NPR homologous amino acid sequences revealed structural similarities in gene structures, conserved motifs, domains, and amino acid residues between PgNPR family members and NPRs of Arabidopsis and rice. PgNPR1-like proteins share structural similarities with NPRs in different plant species, including a BTB/POZ domain at the *N*-terminal, a DUF, Ank conserved domain, and an NPR1-Like C domain at the C-terminal. The BTB/POZ and Ank repeat domains play a crucial role in protein interaction, making them important for its function.^61-63^ The *N*-terminal BTB domain in NPR1 suggests that it may act as a substrate adaptor for CRL3 to degrade an unknown SAR repressor. The C-terminal NLS is necessary for SA-induced nuclear translocation of NPR1, but this alone is not sufficient to induce defense genes, indicating additional activation steps are required in the nucleus.^29^^,^^64^ At the C-terminus, the NIMIN 1/2 binding region is found, which provides probable interaction with SA and thus activates SAR, as NIMIN1 and NIMIN2 are responsive to SA.^65^ According to studies, NPR1-mediated transcription is blocked by its C-terminal contact with NIMIN proteins, which are repressors with an “EAR” (ET-responsive-element-binding-factor-associated amphiphilic repression) motif.^65-67^ SA disturbs this inhibition by triggering a conformational shift in NPR1, obscuring the NIMIN-binding motif.^66^^,^^68^ These results showed that *NPR1-*like genes are highly conserved across a wide range of plant species. From the domain conservation analysis, we found that out of seven *PgNPR1*-like genes, only four, viz. *Pgl_GLEAN_10004488*, *Pgl_GLEAN_10004489*, *Pgl_GLEAN_10007810*, and *Pgl_GLEAN_10029279* had NPR1-like C domains, which are more essential to be identified as a potential *NPR1*-like genes.^69^

 Phylogenetic analysis revealed the evolutionary origins of the *NPR* gene family, where the internal branches of the evolutionary tree represented the relative distances and relationships between *PgNPR1-*like genes and other *NPR* gene family members of wheat, sorghum, rice, barley, and Arabidopsis. Among the seven PgNPR1-like proteins, Pgl_GLEAN_10029279 and Pgl_GLEAN_10007810 were shown to have the closest homology with rice-NPR2 and sorghum-NPR1, respectively. This implies that both the proteins share common ancestors with other NPRs, indicating their similarities in characteristics and functionalities.

The distribution pattern of *NPR1*-like genes in pearl millet indicates that they have undergone chromosomal rearrangements and duplications, suggesting evolutionary modifications during their genetic history. The significant differences in sizes, numbers of introns, and exons observed in the *PgNPR1-*like genes provide compelling evidence of genomic region modifications, including both gene loss and gene gain events, that took place during the expansion of the *NPR1* gene family.

The abundance of stress-responsive elements, such as ARE, MBS, LTR, MYC, MYB, STRE, GC-motif, W-Box, WRE3, WUN-motif, DRE, and TC-rich repeats, within the promoter regions of *PgNPR1*-like genes strongly suggests their essential role in orchestrating and regulating stress response pathways.^70^ Meanwhile, for the seven genes, we detected ACE, AE-BOX, GATA-Box, G-Box, TCT Motif, TGACG Motif, etc., which indicated the involvement of genes in hormone-responsive signals.^71^ These findings suggest that various *PgNPR1-*like genes exhibited involvement in various environments over different time periods and their responses to hormones display intricate patterns.

It is already reported that in Arabidopsis, the NPR1 protein is a receptor for SA, which is an essential hormone in plant defense.^21^ The 3-D structure of two PgNPR1-like proteins, Pgl_GLEAN_10007810 and Pgl_GLEAN_10029279, were obtained (Figure 6A,C). As it is known that NPR1 is a physical receptor of salicylic acid, the structure of SA was also obtained to observe its interaction with the respective proteins. Both the PgNPR1-like proteins were docked with SA, showing that both have strong interactions with SA (Figure 6B,D). The residues involved in the interaction of the salicylic acid with Pgl_GLEAN_10007810 protein were found to be ASN375, GLU459, LYS479, and ALA463, whereas LYS581 and ASP566 were observed to play a vital role in the interaction of salicylic acid with Pgl_GLEAN_10029279. However, in Arabidopsis, CYS521 and CYS529 were found to be crucial for the binding of SA to NPR1.^21^ This provides preliminary information about the binding specificity of SA to NPR1 in different plant species.

While the expression patterns of *NPR1*-like genes in various tissues have been documented in Arabidopsis, wheat, potatoes, pears, and mustard, little is known about how these genes express themselves in pearl millet tissues.^55^^,^^57^^,^^71^^-^^73^ In this study, transcript profiling was performed through qPCR in six different tissues of pearl millet. *Pgl_GLEAN_10029279* was shown to have constant expression levels across all tissues, with the greatest levels found in seed samples. It implies that *Pgl_GLEAN_10029279* may have contributed to organ symmetry and determinacy during leaf morphogenesis. Strong expressions of *Pgl_GLEAN_10015079*, *Pgl_GLEAN_10004489*, and *Pgl_GLEAN_10007810* were found in the root samples, but weak expression was detected in the seed samples. In the stem tissues, *Pgl_GLEAN_10004489* and *Pgl_GLEAN_10015079* had significantly greater expression levels. In flag leaf tissues, *Pgl_GLEAN_10004489* was more expressed compared to other genes. Significant expression of *Pgl_GLEAN_10007810* and *Pgl_GLEAN_10015079* was detected in the inflorescence tissues, which might imply their possible involvement in the early flower development. These findings highlight the potential role of *PgNPR1*-like genes in coordinating developmental events, reacting to environmental stresses, and regulating essential activities. Transcripts were not detected at all for the genes *Pgl_GLEAN_10004488*, *Pgl_GLEAN_10033256*, and *Pgl_GLEAN_10027009* in any of the tissue samples, which suggests their probable involvement in some other physiological processes of *P. glaucum*.

 SA and MeJA play crucial roles in plant disease resistance and defensive responses. The SA and MeJA signaling pathways are linked and cooperative. NPR1, a critical regulatory element, functions as both an SA receptor and a positive regulator of systemic acquired resistance (SAR), playing an important role in SA/MeJA signaling. According to previous studies, the SA pathway inhibits JA-regulated genes, a function that requires NPR1.^74^ We discovered that *Pgl_GLEAN_10029279* and *Pgl_GLEAN_10007810*, two of the seven identified *PgNPR1*-like genes, interact significantly with the SA ligand. The transcriptional expression study of these genes following SA and MeJA treatment revealed substantial changes in expression patterns. The expression of *Pgl_GLEAN_10029279* varied across the 2, 12, 24, and 48-h time points following salicylic acid treatment, showing no clear or consistent temporal pattern. However, after 2, 12, 24, and 48 h of MeJA treatment, *Pgl_GLEAN_10029279* was shown to be significantly downregulated. For the other gene, *Pgl_GLEAN_10007810*, we observed no expression after treatment with SA and MeJA at time points of 2, 12, 24, and 48 hours of treatment. In later time points, such as 3 and 5d post treatment with SA and MeJA, there was no significant expression observed in comparison with the control samples. The variable expression of the gene under these hormonal stress conditions over different time points suggests a complex response in the regulatory network controlling plant responses to biotic stresses. In addition, promoter analysis of *Pgl_GLEAN_10029279* revealed the presence of several cis-regulatory elements, including as−1, MeJARE, TATA-box, Myb, ABRE, STRE, LTR, TC-rich, LRE, Sp1, WRE, and DRE motifs (Supplementary Figure S4). The presence of these cis-regulatory elements, especially SA, MeJA, and abiotic stress-responsive elements, along with the expression patterns of *Pgl_GLEAN_10029279* under these treatments and stress conditions, provides insights into its involvement in various biotic and abiotic stress responses. Though SA and MeJA signaling mechanisms are antagonistic in nature, we could not find related expression, which indicates that the dynamic and context-dependent regulation of NPR1, being influenced by the cellular redox state, feedback loops, and interactions with other signaling pathways.^75^ It shows the involvement of the *NPR1*-like genes in intricate hormone signaling during the defense mechanism of pearl millet.

Fungal diseases such as blast (*M. grisea*) and downy-mildew (*S. graminicola*) are the two of the most dangerous diseases that affect pearl millet. Blast contributed to nearly 15% pearl millet yield loss.^76^ Downy-mildew infection has been linked to a disease incidence of 45%–95% and a yield loss of 20%–80% over reports.^77^^,^^78^ This work examined the expression profile of the *PgNPR1*-like genes after treatment with *M. grisea* and *S. graminicola*. After *M. grisea* infection, only *Pgl_GLEAN_10029279* was expressed among the seven *PgNPR1*-like genes. *Pgl_GLEAN_10029279* revealed considerable overexpression after 3 d post-inoculation with *M. grisea*. However, the expression pattern showed 3 and 5 d after SA and MeJA treatments does not align with this result. No detectable expression was found at these later time points, most likely as a result of active chemical being lost by volatilization, wash-off, or metabolic deactivation. In contrast, pathogen infection prolongs gene induction and sustains endogenous hormone synthesis. Previously, transcriptome sequencing of genes associated with pearl millet–downy-mildew interactions was performed by Kulkarni et al.^53^ In this report, pearl millet susceptible (7042S) and resistant (P310-7) varieties were used for transcriptome sequencing upon *S. graminicola* pathogen challenge, and submitted with SRA database accession numbers viz. SRX885597, SRX1885493, SRX1885494, and SRX1885495. In transcriptome datasets, the expression of *Pgl_GLEAN_10004489* and *Pgl_GLEAN_10029279* was significantly upregulated in the resistant varieties upon pathogen infection. Moreover, for *Pgl_GLEAN_10004489*, the basal level expression of the gene was higher in the resistant variety than in the susceptible one. It shows that both genes might have potential roles against downy-mildew infection.

Although NPR1 is known to be a transcriptional coactivator in plant defense against phytopathogens,^79^ but its role under abiotic stress has not been well understood. It has been reported that NPR1 has a crucial role in ionic homeostasis in Arabidopsis roots during salt stress but not in salt tolerance.^31^^,^^32^ Our study revealed that *Pgl_GLEAN_10029279*, *Pgl_GLEAN_10004489*, and *Pgl_GLEAN_10015079* were significantly upregulated after 12 h of salinity stress, which indicates the possible positive regulatory roles of these *PgNPR1*-like genes in maintaining ionic homeostasis in pearl millet. This result also has a similar pattern with already reported NPR members in *Brassica juncea*.^71^ According to a recent study, chloroplast NPR1 acts as a retrograde transmitter of protective machinery to the nucleus via redox reaction, particularly in photosynthetically active leaf cells under salt stress, and because the majority of *PgNPR1*-like genes, identified in this study, are predicted to be localized in chloroplasts, they are likely to show significant upregulation after salinity stress.^80^ SA-induced NPR1 condensates may promote stomatal closure and cell viability during drought stress. Drought has been linked to increased NPR1 expression, which promotes ROS production and stomatal closure.^81^^,^^82^ This information correlates with our findings, where we observed induced expression for *Pgl_GLEAN_10029279*, *Pgl_GLEAN_10004489*, and *Pgl_GLEAN_10015079* after drought treatment. This result provides information about the possible involvement of the *PgNPR1*-like genes in pearl millet defense. Under the heat treatment, *Pgl_GLEAN_10029279* and *Pgl_GLEAN_10004489* exhibited very little downregulation, whereas *Pgl_GLEAN_10004488* showed considerable upregulation. Heat stress in plants produces ROS, which impairs plant growth and acclimatization.^83^ So, it is difficult to comprehend the roles of the mentioned *PgNPR1*-like genes in heat stress, but there is a possibility that these genes are involved in the regulation of ROS production. Further studies are needed to observe the appropriate roles of *PgNPR1*-like genes under heat stress.

In our study, we found that three of the *PgNPR1-*like genes, *Pgl_GLEAN_10007810*, *Pgl_GLEAN_10033256*, and *Pgl_GLEAN_10015079,* were located at the telomeric regions of chromosomes 6, 4, and 1, respectively. The low-level expression of the three genes in the tissue-specific analysis, as well as after treatment with SA and MeJA, may suggest the probable presence of the specific genes at the telomeric heterochromatin of the mentioned chromosomes.

The activation of immunity against pathogens in plants is a challenging process because it requires the involvement of numerous immune mechanisms. Numerous disease-resistant genes and pathogenesis-associated proteins have been identified to enhance the plant's defense mechanisms against a wide range of diseases. SA is a key signaling molecule in plant defense responses, particularly in the activation of SAR. NPR1 acts downstream of SA signaling and is a critical mediator of SA-induced gene expression. It interacts with SA and controls the activation and nuclear translocation of defense-related transcription factors, thereby orchestrating the transcriptional reprogramming necessary for effective pathogen defense. The translation of the *PR* gene is regulated by the TGA family of bZIP transcription factors called NPR1-interacting proteins (NIPs), in conjunction with the *as-1-like* (TGACG) element found in the promoter region of the *PR* gene.^84^^,^^85^ Identifying *NPR1* genes in plants is crucial for elucidating the intricacies of plant defense mechanisms, enhancing crop resilience against diseases, and deepening our comprehension of plant-pathogen interactions. Combining *in silico* analysis of the discovered *PgNPR1*-like genes with their transcriptional profiles would aid in selecting potential *PgNPR1*-like genes for defining their functional significance in the pearl millet's biotic and abiotic stress tolerance mechanism.

Similar research on *AtNPR1* overexpression in rice, particularly in terms of increased resistance to bacterial blight with no negative impacts on agronomic characteristics or fertility, should be performed for pearl millet NPR1.^86^ Understanding how NPR1 regulation influences disease resistance in pearl millet may lead to the development of techniques for increasing its tolerance to biotic stressors, perhaps without sacrificing other desired features. This study might open the way for the use of NPR1-mediated processes in molecular breeding efforts to improve pearl millet resistance to diverse diseases.

## Conclusions

In this research, we have identified seven *PgNPR1-like* genes based on genome-specific data. Phylogenetic studies, structural analysis like conserved domain, conserved motif, promoter analysis, and 3-dimensional hypothetical structural prediction confirmed the initial identification of all the putative *PgNPR1*-like genes. Expression profiling of the seven identified genes under SA and MeJA treatments confirmed the probable involvement of *Pgl_GLEAN_10029279* in plant defense mechanisms. *Pgl_GLEAN_10029279* was observed to be significantly induced by *M. grisea* biological stress conditions. The *PgNPR1*-like genes have shown a differential expression pattern after infection with *S. graminicola*. *NPR1*-like gene family members showed notable variations in gene expression analyses of *P. glaucum* during salt, drought, and heat stress, particularly in *Pgl_GLEAN_10015079*, *Pgl_GLEAN_10004488*, *Pgl_GLEAN_10004489,* and *Pgl_GLEAN_10029279*. These results contribute significantly to our knowledge of the functions and evolution of *NPR1*-like genes in pearl millet, along with their function in metabolic and physiological pathways. Understanding the underlying principles of plant disease resistance, particularly the involvement of the NPR1 protein, is quite interesting. NPR1 is crucial for increasing tolerance to biotic (and occasionally abiotic) problems, whether through classic NPR1 allele breeding or transgenic methods. Identifying particular, more active alleles or combining NPR1-related genes may accelerate molecular breeding efforts.^86^ Candidate PgNPRs can also be used for crop enhancement using genome editing tools and molecular breeding methods to increase agricultural output and guarantee future food security.

## Authors' contributions

JN: bioinformatic analysis, expression analysis, and writing; JC: bioinformatic analysis; BS: formal analysis and editing; SRM, and NM: molecular docking; ND, and MG: conceptualization, validation, and editing; all authors contributed to the article and approved the submitted version.

## Supplementary Material

Supplementary material Table S1: Presence of domains in all seven identified* PgNPR1*-like genes.

Supplementary material Figure S1. Multiple sequence alignment of identified full-length PgNPR1 proteins obtained with ClustalW and manual correction compared with other known NPR1-like sequences, i.e., Arabidopsis NPR1 and Rice NPR5. The shaded colors indicate low to high amino acid residue conservation, i.e., blue to red. The conserved domains BTB/POZ and ANK, important motifs, the NIMIN-binding region, and the nuclear localization signal (NLS) are highlighted with solid lines.

Supplementary material Figure S2. Semiquantitative RT‒PCR results of endogenous control *GAPDH* and *Pgl_GLEAN_10007810* with salicylic acid (SA) and methyl jasmonate (MeJA) treated samples.

Supplementary material **Fig S3.** The transcriptional expression patterns of *Pgl_GLEAN_10029279* under (**A)** Salicylic Acid treatment, (**B)** Methyl Jasmonate treatment. The transcriptional expression pattern was analysed by RT-qPCR. EF1α and was used as the internal reference gene. The data shown are representative of three independent biological replicates, and all data points indicated the mean ± standard error (SE) of the three biological repeats.

Supplementary material **Fig.S4** Schematic representation of cis-regulatory elements identified in the promoter region of Pgl_GLEAN_10029279. The horizontal black line indicates the promoter backbone, and colored boxes represent individual cis-elements positioned according to their location (bp) relative to the transcription start site. Cis-elements are color-coded according to their type, including as-1, MeJARE, TATA-box, Myb, ABRE, STRE, LTR, TC-rich, LRE, Sp1, WRE, and DRE motifs, which are associated with diverse transcriptional responses such as hormonal regulation, light signaling, and abiotic or biotic stress adaptation.

Supplementary material **Semiquantitative PCR conditions:** **Quantitative PCR conditions:**

Supplementary material Table S2: Sequence characteristics and chromosomal coordinates of identified NPR members in pearl millet.

## Data Availability

Additional data supporting the findings of this study are available upon request.
