# Supplementary material for: Genome-wide identification and expression analysis of NPR1-like genes in pearl millet under diverse biotic and abiotic stresses and phytohormone treatments
Source: Plant Signal Behav. 2025 Sep 7;20(1):2552895. doi: 10.1080/15592324.2025.2552895 (PMC12427447; doi:10.1080/15592324.2025.2552895)
Supplement: Supplementary material — Figure S1. Multiple sequence alignment of identified full-length PgNPR1 proteins obtained with ClustalW and manual correction compared with other known NPR1-like sequences, i.e., Arabidopsis NPR1 and Rice NPR5. The shaded colors indicate low to high amino acid residue conservation, i.e., blue to red. The conserved domains BTB/POZ and ANK, important motifs, the NIMIN-binding region, and the nuclear localization signal (NLS) are highlighted with solid lines. [file KPSB_A_2552895_SM2761.docx]

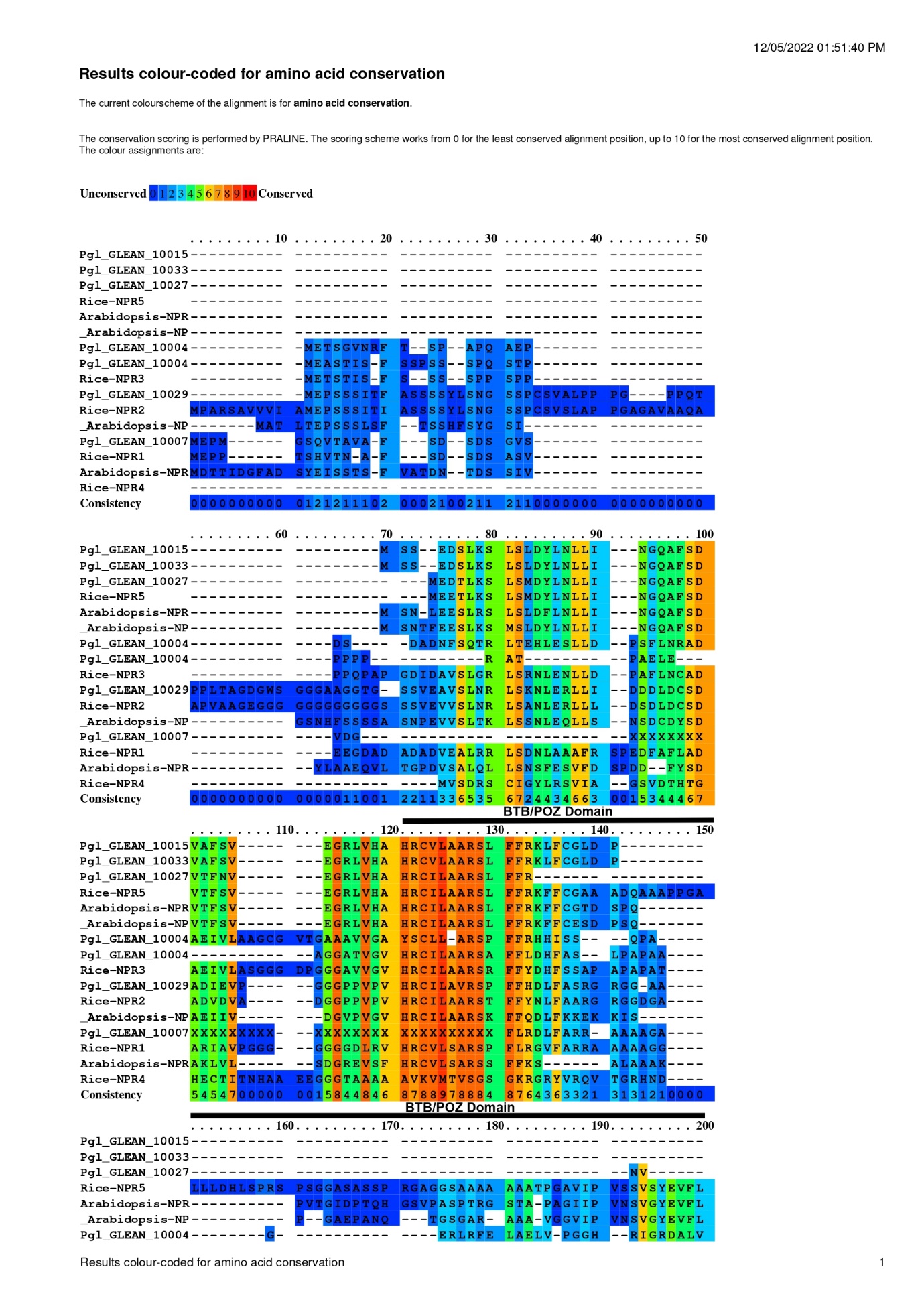

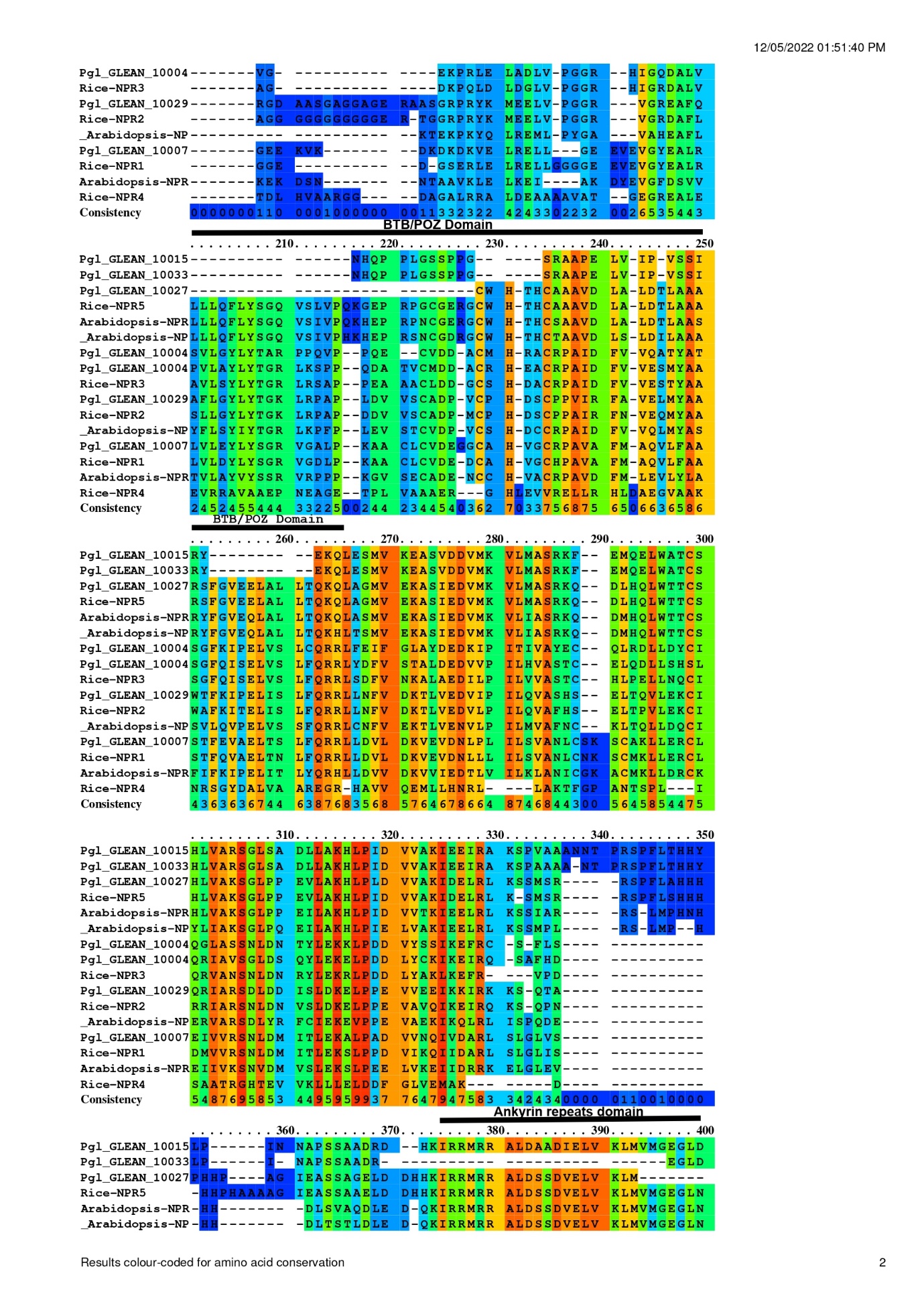

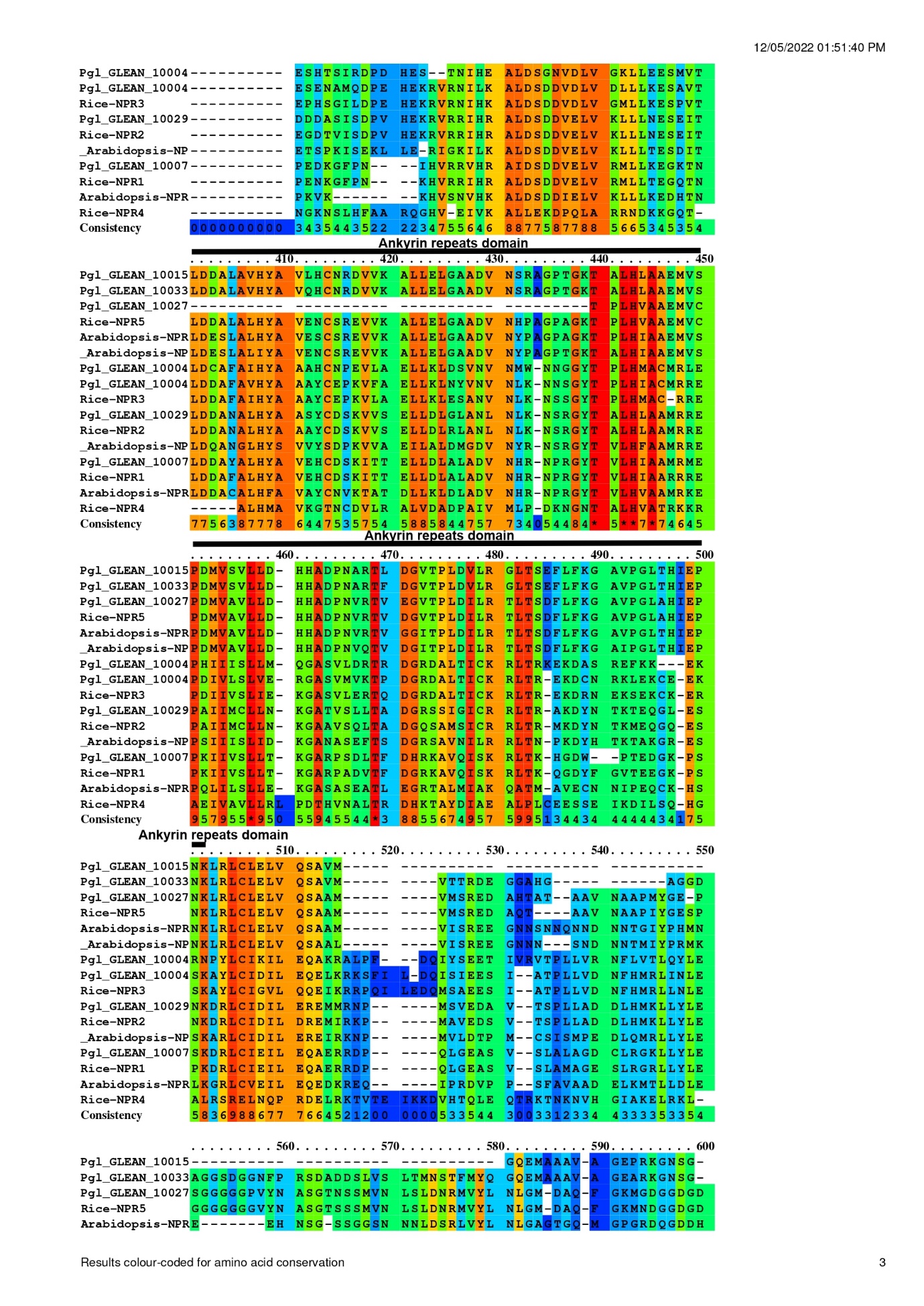

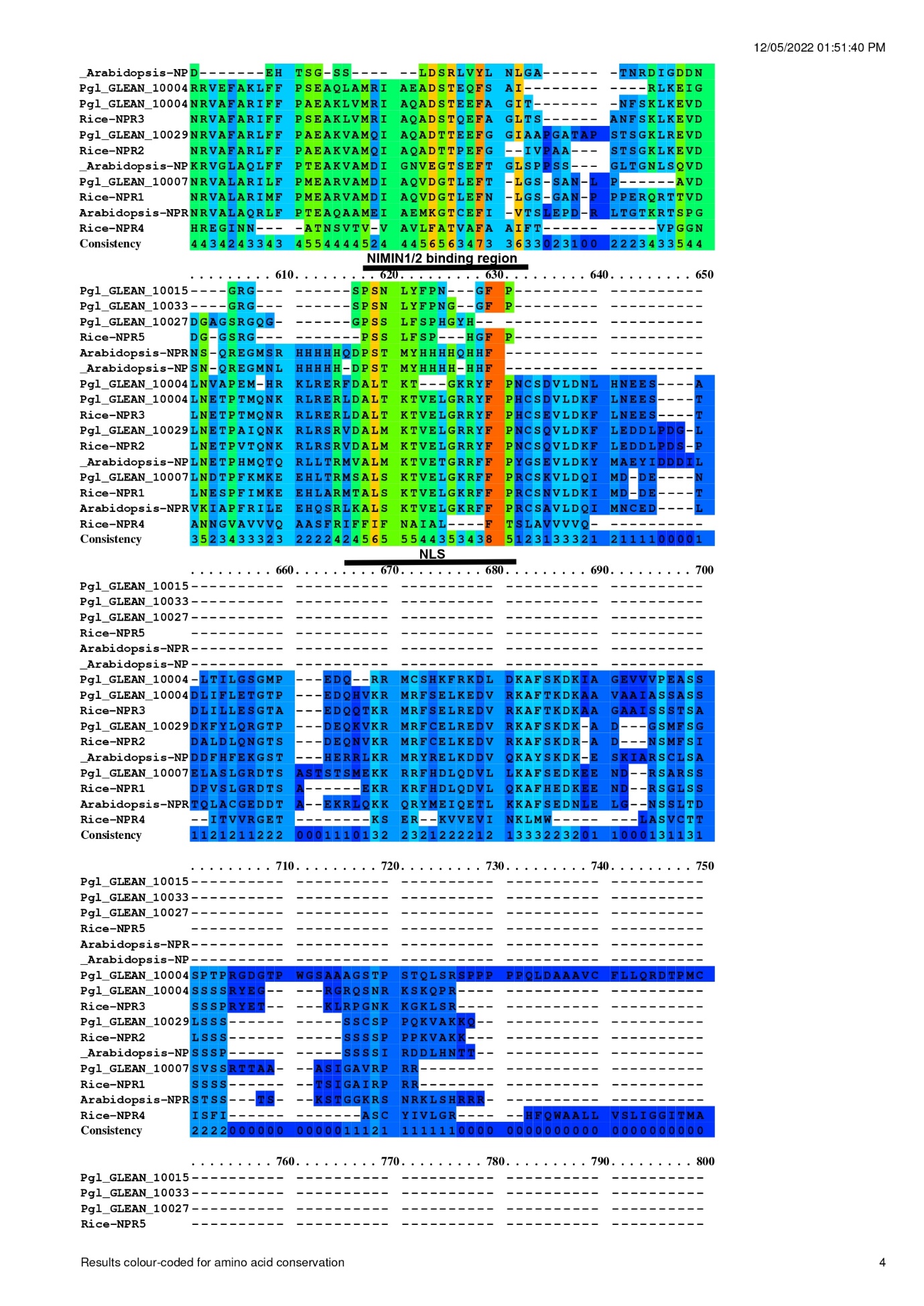

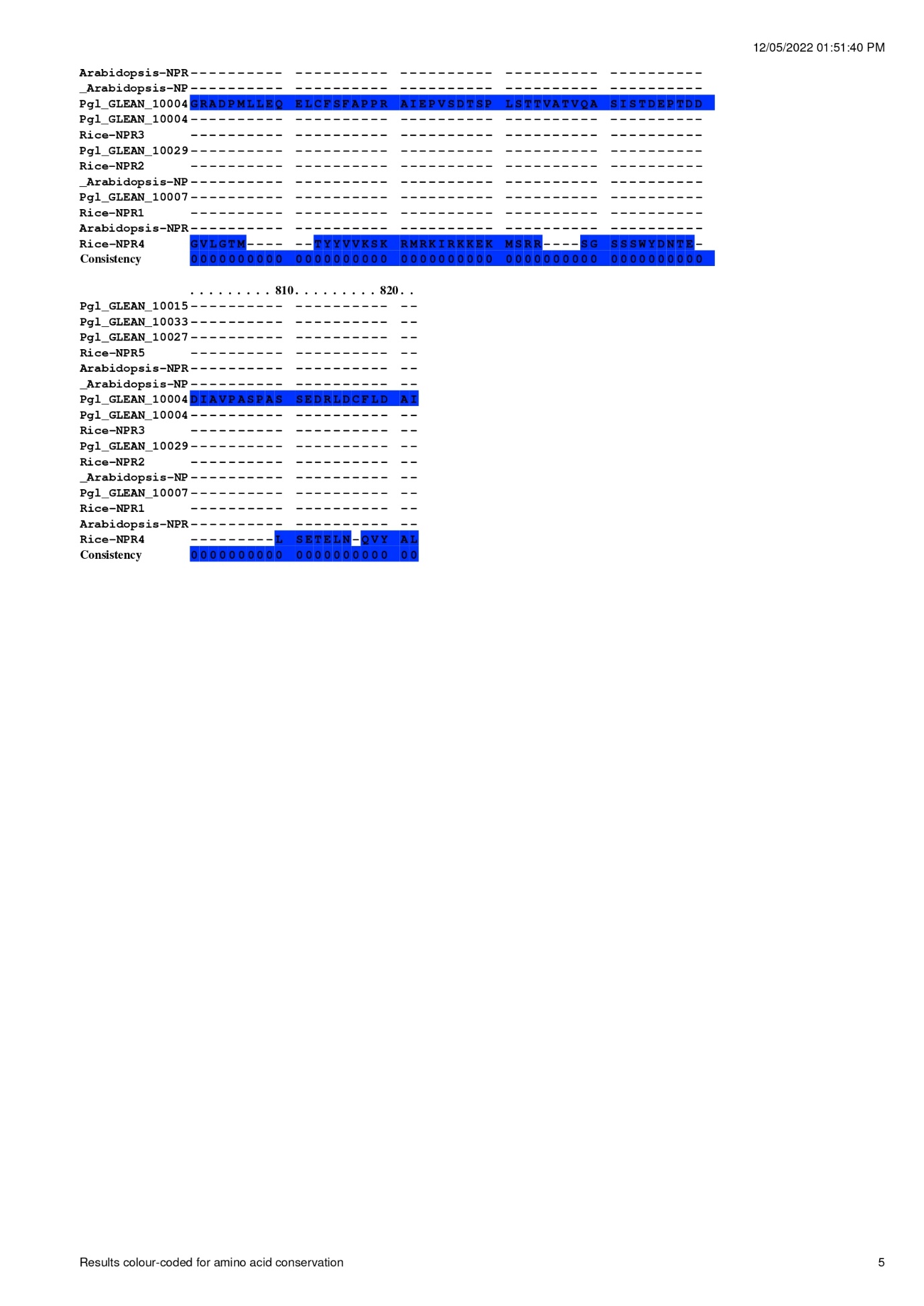


Fig.S1 Multiple sequence alignment of identified full length PgNPR1 proteins obtained with ClustalW and manual correction, compared with other known NPR1-like sequences i.e. Arabidopsis NPR1 and Rice NPR5. The shaded colours indicate low to high amino acid residue conservation i.e., blue to red. The conserved domains, BTB/POZ and ANK, and important motifs, NIMIN-binding region, and nuclear localization signal (NLS), are highlighted with solid lines.
