# Supplementary material for: Genome-wide identification and expression analysis of NPR1-like genes in pearl millet under diverse biotic and abiotic stresses and phytohormone treatments
Source: Plant Signal Behav. 2025 Sep 7;20(1):2552895. doi: 10.1080/15592324.2025.2552895 (PMC12427447; doi:10.1080/15592324.2025.2552895)
Supplement: Supplementary material — Figure S2. Semiquantitative RT‒PCR results of endogenous control GAPDH and Pgl_GLEAN_10007810 with salicylic acid (SA) and methyl jasmonate (MeJA) treated samples. [file KPSB_A_2552895_SM2762.docx]

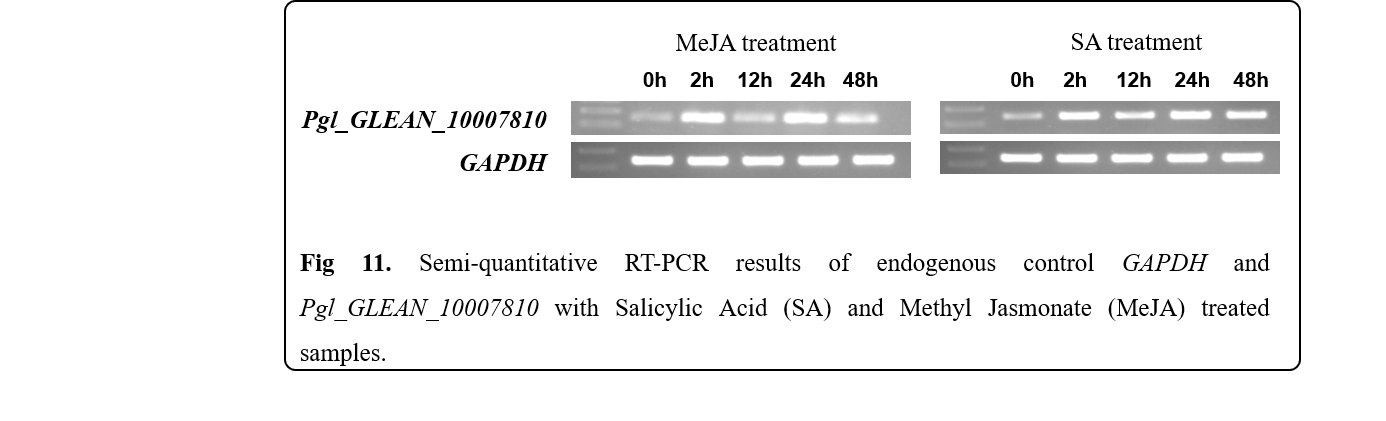


Fig.S2. Semi-quantitative RT-PCR results of endogenous control *GAPDH* and *Pgl_GLEAN_10007810* with Salicylic Acid (SA) and Methyl Jasmonate (MeJA) treated samples.
