# Supplementary material for: Genome-wide identification and expression analysis of NPR1-like genes in pearl millet under diverse biotic and abiotic stresses and phytohormone treatments
Source: Plant Signal Behav. 2025 Sep 7;20(1):2552895. doi: 10.1080/15592324.2025.2552895 (PMC12427447; doi:10.1080/15592324.2025.2552895)
Supplement: Supplementary material — Fig S3. The transcriptional expression patterns of Pgl_GLEAN_10029279 under (A) Salicylic Acid treatment, (B) Methyl Jasmonate treatment. The transcriptional expression pattern was analysed by RT-qPCR. EF1α and was used as the internal reference gene. The data shown are representative of three independent biological replicates, and all data points indicated the mean ± standard error (SE) of the three biological repeats. [file KPSB_A_2552895_SM3183.docx]

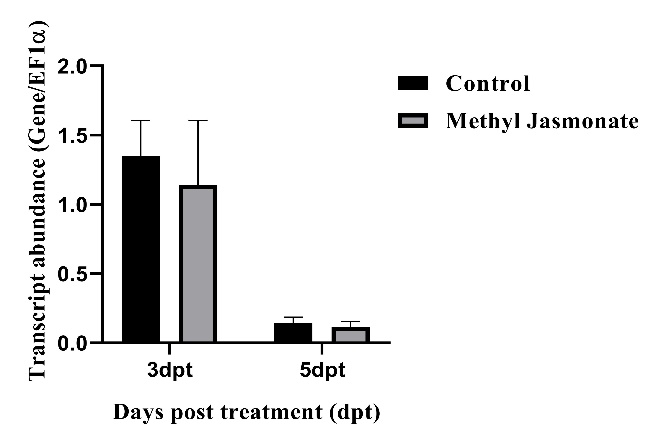

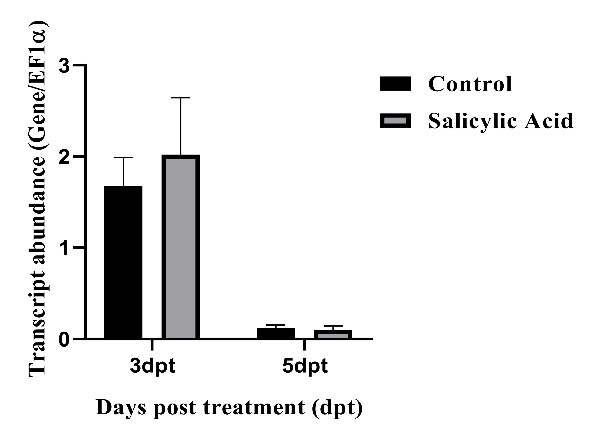


**B)**

**A)**

**Fig S3** The transcriptional expression patterns of *Pgl_GLEAN_10029279* under (**A)** Salicylic Acid treatment, (**B)** Methyl Jasmonate treatment. The transcriptional expression pattern was analysed by RT-qPCR. EF1α and was used as the internal reference gene. The data shown are representative of three independent biological replicates, and all data points indicated the mean ± standard error (SE) of the three biological repeats.
