# Supplementary material for: Genome-wide identification and expression analysis of NPR1-like genes in pearl millet under diverse biotic and abiotic stresses and phytohormone treatments
Source: Plant Signal Behav. 2025 Sep 7;20(1):2552895. doi: 10.1080/15592324.2025.2552895 (PMC12427447; doi:10.1080/15592324.2025.2552895)
Supplement: Supplementary material — Fig.S4 Schematic representation of cis-regulatory elements identified in the promoter region of Pgl_GLEAN_10029279. The horizontal black line indicates the promoter backbone, and colored boxes represent individual cis-elements positioned according to their location (bp) relative to the transcription start site. Cis-elements are color-coded according to their type, including as-1, MeJARE, TATA-box, Myb, ABRE, STRE, LTR, TC-rich, LRE, Sp1, WRE, and DRE motifs, which are associated with diverse transcriptional responses such as hormonal regulation, light signaling, and abiotic or biotic stress adaptation. [file KPSB_A_2552895_SM3186.docx]

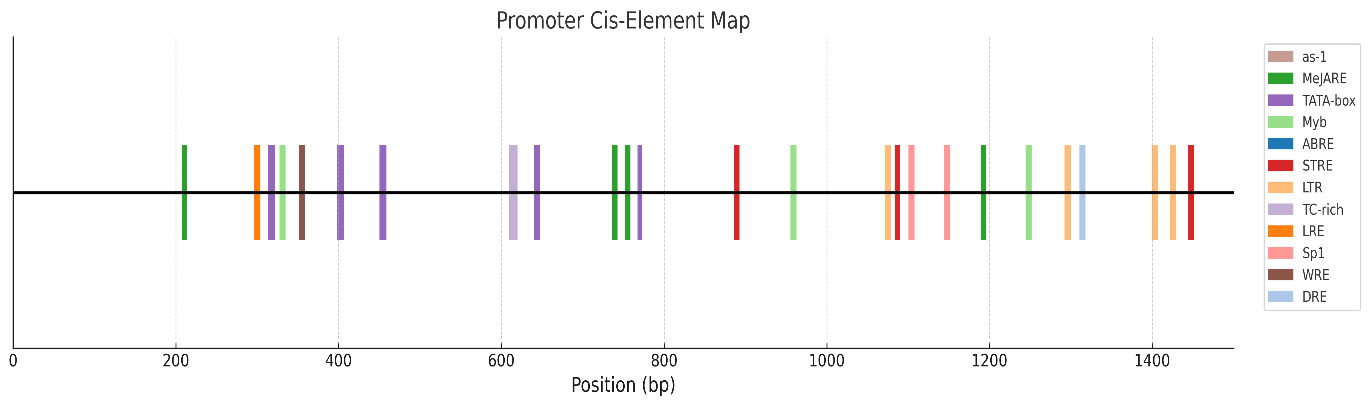


**Fig.S4** Schematic representation of cis-regulatory elements identified in the promoter region of Pgl_GLEAN_10029279. The horizontal black line indicates the promoter backbone, and colored boxes represent individual cis-elements positioned according to their location (bp) relative to the transcription start site. Cis-elements are color-coded according to their type, including as-1, MeJARE, TATA-box, Myb, ABRE, STRE, LTR, TC-rich, LRE, Sp1, WRE, and DRE motifs, which are associated with diverse transcriptional responses such as hormonal regulation, light signaling, and abiotic or biotic stress adaptation.
