# Supplementary material for: Genome-wide identification and expression analysis of NPR1-like genes in pearl millet under diverse biotic and abiotic stresses and phytohormone treatments
Source: Plant Signal Behav. 2025 Sep 7;20(1):2552895. doi: 10.1080/15592324.2025.2552895 (PMC12427447; doi:10.1080/15592324.2025.2552895)
Supplement: Supplementary material — Semiquantitative PCR conditions: Quantitative PCR conditions: [file KPSB_A_2552895_SM2763.docx]

| **Gene ID names** | **Primer Sequences** |
| --- | --- |
| *Pgl_GLEAN_10004488* | FP- GTTGAGAGAACGTTTTGATG  RP- GTATGGTCAAAGCAGATTCT |
| *Pgl_GLEAN_10004489* | FP- GAAGGAGGTTGATCTAAAT  RP- GAACAATGTGGGAAGTAC |
| *Pgl_GLEAN_10007810* | FP- TGCTCTACCTTGAAAATC  RP- GTCATTTAGATCAACAGCA |
| *Pgl_GLEAN_10015079* | FP- ACCTCCGAGTTCCTCTTCAA  RP- AAGTACAAGTTGGAGGGGCT |
| *Pgl_GLEAN_10027009* | FP- GACGACCACCACAAGATCC  RP- ATGTCTGGGCACACCATC |
| *Pgl_GLEAN_10029279* | FP- ACTTCTGGGAAACTGAGGGA  RP- AAGCGCATCCTCTTCACCTT |
| *Pgl_GLEAN_10033256* | FP- TCTTCTTCCGCAAGCTCTTC  RP- CCTTCACCATGCTCTCCAG |

**Semiquantitative PCR conditions:**

| **Step** | **Temperature** | **Time** | **Cycles** |
| --- | --- | --- | --- |
| Initial denaturating | 95°C | 2 min | 1 |
| Denaturating | 95°C | 30 sec | 35 |
| Annealing | 50-65°C | 30 sec |  |
| Extension | 72°C | 30 sec |  |
| Final Extension | 72°C | 5 min | 1 |

**Quantitative PCR conditions:**

| **Step** | **Temperature** | **Time** | **Cycles** |
| --- | --- | --- | --- |
| Initial denaturating | 95°C | 10 min | 1 |
| Denaturating | 95°C | 15 sec | 40 |
| Annealing | 58°C | 1 min |  |
| Extension | 72°C | 30 sec |  |
